# Supplementary material for: Chromothripsis during telomere crisis is independent of NHEJ, and consistent with a replicative origin
Source: Genome Res. 2019 May;29(5):737–49. doi: 10.1101/gr.240705.118 (PMC6499312; doi:10.1101/gr.240705.118)
Supplement: Supplemental Material [file supp_gr.240705.118_Supplemental_file_1.zip › contigs/annotated_contigs/DB38/contig.3.DB38_length_516_mean_cov_8.7015503876.docx]

**DB38_length_516_mean_cov_8.7015503876**

```
TGTAAGCACATGAAAAGATGTTTAACATTAATGGAATGTTTGATTTGCCTCAGAGAACTACAAGTCAAAACTACAATGAGACTCTACTT
 >chr4:185959716-185959968 - E=3e-135 p=2e-02
CACACCCATTAGAATAGCTGTTATTTAAAAAAAAAAAAGCAGAAAATAACAAGTGTTGGTGAGGATATGGAGAAACTGGAACCCTCTGC

ATTGATGGTGGGAATATAAAATGGCACAGCCATTGCGGAAAACAGTATGGGGGTTCCTCAAAAAATTAAACA|T|TATTTCATTTAGCA
                                                                         >chr4:18595944
TAATTTCTTTAAGATCATCCAGGTTGTAGTATCACAATTTCCTTCCTTTTCAAGGCTGAATAATATTCCATTGTATTTATATACCACAT
3-185959589 + E=3e-76
TTTATTTATCCATTTATCCATTAATGGACACTTAGGAGGTTT|AAA|CAAGGTAGCAGGTGCAGAGGCAGCAATGTGGGAAGCAGAAAG
                                              >chr4:185958056-185958173 - E=9e-59
CCACGAGGGTAGGGAGGAGGGCAAGAGTGGAGCACAGTCCTGTCGCTGTGGGGAGGAGCTTGGGTTTAACCTGCA
```
